# COVID-19 Pandemic: The Impact of COVID-19 on Mental Health and Life Habits in the Canadian Population

**DOI:** 10.3389/fpsyt.2022.871119

**Published:** 2022-06-29

**Authors:** Felicia Iftene, Roumen Milev, Adriana Farcas, Scott Squires, Daria Smirnova, Konstantinos N. Fountoulakis

**Affiliations:** ^1^Providence Care Hospital, Department of Psychiatry, Queen’s University, Kingston, ON, Canada; ^2^Center for Neuroscience Studies, Queen’s University, Kingston, ON, Canada; ^3^International Centre for Education and Research in Neuropsychiatry (ICERN), Samara State Medical University, Samara, Russia; ^4^3rd Department of Psychiatry, School of Medicine, Aristotle University of Thessaloniki, Thessaloniki, Greece

**Keywords:** depression, suicidality, mental health, conspiracy theories, COVID-19, Canada, lockdown

## Abstract

**Objectives:**

The study aims to investigate the rate of clinical depression in the adult population during the COVID-19 pandemic, as well as the changes in anxiety, distress, suicidal ideation, and their relations with several personal and interpersonal/social variables.

**Methods:**

This is an epidemiological, non-interventional study. It is part of an international multi-center study, with the main site at the Aristotle University of Thessaloniki, in Greece (COMET-G Study). We are presenting aspects of the research involving the Canadian site, based on 508 Canadian responders to the online survey (QAIRE).

**Results:**

Of the 508 responders, 72.2% were females aged 42.57 ± 14.00 years; 27.2% were males aged 42.24 ± 15.49 years; and 0.6% were others aged 46.33 ± 17.79 years. Increased anxiety during the lockdown was reported by 69.3% of those surveyed. The rate of suicidal thoughts increased in 19.5% of participants during the lockdown. Depression was reported by 22% of responders, while distress was present in 18.4%. We found a greater prevalence of depression, but not distress, in individuals with a history of any mental disorder. Based on the multiple regression analysis, we found four CORE factors equally influencing the changes in mental health during the lockdown (gender, quality of sleep, family conflicts, and changes in daily routine). In the Canadian population, two major changes acted as protective factors, significantly expressed when compared with the worldwide tendencies: fewer financial difficulties; and an increase in religious beliefs.

**Conclusion:**

The rate of major depression, distress, and suicidal ideation was higher in Canadians than in the worldwide population (per COMET-G), but the relative risk to develop depression in the presence of a history of mental disorders was lower. Almost 90% of Canadians believed in the real story of COVID source of provenience.

## Introduction

It has been more than 2 years since March 11, 2020, when the COVID-19 pandemic was declared. We started our journey through a very difficult time of uncertainty, facing an extreme threat. We have been dealing with major changes in our daily routines and expectations, facing isolation, unemployment, economic crisis, and increased human losses. We tried to better understand “the enemy,” to get organized, develop, and follow rules and protocols. According to Statistics Canada, 90% of Canadians adopted public health precautions in less than 1 month.

In December 2020, during the second lockdown in Ontario and Alberta, the COVID vaccination started. In January 2022, during the Omicron wave, 84.3% of the Canadian population aged 5 years or older had received their first dose of vaccine (83.48% of Ontarians), while 78.0% were fully vaccinated. In Ontario, where we had one of the most successful vaccination campaigns in the world, 91.2% of vaccine doses have been administered (1).

Regarding the coping aspect of this pandemic, in June 2021, only 55% of Canadians reported “excellent” or “very good” mental health, compared with 68% in 2019. As expected, different nuances of anxiety and depression were experienced, going from extremis to panic and desperation.

There is a large body of literature analyzing different aspects of mental health changes during the COVID-19 pandemic, in general, as well as during lockdowns, in particular (2–5). A recent study, which included 1,006 subjects from Italy, evaluated the psychological repercussions of isolation during the first lockdown in the spring of 2020, concluding that the longer the isolation and the less adequate the physical space where people were isolated, the worse the mental health was (e.g., depression); during this critical time, online contact was found to be crucial in protecting mental health (6). A longitudinal observational study from Germany (7) focusing on anxiety disorders suggests that those with a personal history of mental disorders, particularly anxiety disorders and generalized anxiety disorder, are vulnerable to experience psychological strain in the context of the pandemic; they might likely overestimate the potential threat and should be targeted by preventive and therapeutic interventions. Using qualitative methods (8), it was identified that a history of anxiety or depression affects multiple levels of the social-ecological model during the pandemic, defining the most important mental health stressors. At the individual level, we see isolation/loneliness, fear of contracting COVID-19, and uncertainty about the future; at the interpersonal level, we have fears of family members contracting COVID-19, separation from family members, and domestic relationships; while at the community and societal level, these stressors are represented by employment, community and societal systems, and media. At the beginning of the pandemic, an Italian study (9), aimed at identifying psychological changes during the lockdown period as well as factors associated with these changes, was conducted (original sample = 2,766, with an online follow-up survey for 439 participants). The authors found an increase in stress and depression over the lockdown, but not anxiety. Higher levels of depression at the start of the lockdown, as well as fewer coping strategies and childlessness, were associated with increased depression at follow-up, whereas higher levels of stress at the start of the lockdown and younger age were associated with higher stress at follow-up. In China [Le (10)], a large sample, cross-sectional, population-based, online survey study was conducted to investigate the prevalence of and risk factors associated with mental health symptoms in the general population (56,679 participants), during the COVID-19 pandemic. The results of this survey indicate that mental health symptoms may have been common during the COVID-19 outbreak, especially among infected individuals, people with suspected infection, and people who might have contact with patients with COVID-19. Some measures, such as quarantine and delays in returning to work, were also associated with a decline in mental health among the public.

The COVID-19 pandemic stormed our lives and changed our perspectives. Unfortunately, studies on COVID-19 have significant data heterogeneity, largely due to differing cultural/economic specifics between countries, as well as differences in timing (data collected in different phases of the pandemic), sample size, and instruments. There was a need for a holistic, longitudinal, and comparable, real-time assessment of emotional, behavioral, and societal impacts of the COVID-19 pandemic across nations (11, 12). Konstantinos Fountoulakis and his international research team worked on the COMET-G study, which gives a larger picture of the phenomena by using both a standard time frame and identical instruments in 40 counties across the world. Our current paper is a part of Fountoulakis’ international study, specifically looking at the mental health changes and their possible determinants in Canadians.

As researchers and mental health providers within the scope of precision and personalized psychiatry, we consider it important to identify the exact contribution of specific variables to observed pathology. This research aims to assess how the COVID-19 pandemic, either in itself or as a result of the measures adopted to control the outbreaks, has affected various aspects of the mental functioning, needs, and behaviors of the general population.

We are referring here only to the Canadian part of the research, based on 508 Canadian responders to the online survey. This portion of the international study aims to investigate the rate of clinical depression in the adult population over the age of 18 years in Canada during the COVID-19 pandemic. Secondary aims are to investigate the changes in anxiety, distress, suicidal ideation, and their relations with several personal and interpersonal/social variables.

We hypothesized that there will be an increase in depression and distress across the general Canadian population, with a higher rate in those with a history of any mental condition.

## Materials and Methods

This is an epidemiological, non-interventional study – part of an international multi-center study, with the main site at the Aristotle University of Thessaloniki, Greece, Europe. The questionnaire used for this project was developed as part of an initiative by the Mental Health Sector of the Scientific Researches Institute of the Pan-Hellenic Medical Association.

This study was started in Europe, and the initial approval for this research project was given by the Ethics Committee of the Faculty of Medicine, Aristotle University of Thessaloniki, Greece. For our part of this research, we received approval from the Health Science Research Ethics Board at Queen’s University, Kingston, Canada. Participation in this study was voluntary and the respondents had to be at least 18 years old to participate. The first QAIRE page included a declaration of consent, which all participants accepted before filling out the survey. Due to the format of the questionnaire, respondents were required to have access to technology and be able to read and answer questions in English or French.

We used two types of Google Documents links: one with the questionnaire for the respondents and one for monitoring purposes for the research team.

For data collection, the Google Documents (one in English and another in French) disseminated the QAIRE anonymous questionnaire across the general population of Canada. QAIRE was designed as an anonymous research tool (each respondent provided only their year of birth, with no other personal data being required or registered). Using a separate link, the research team was able to use Google Documents to check the number of responses and to facilitate the data collection, when necessary. This link was only for use in Canada, as the questionnaire related to Canada’s lockdown conditions (and not any other country). All responses were automatically saved and summarized in the associated Excel file on Google Disc. Only Prof. Konstantinos Fountoulakis, two general coordinators, and our research team had access to this dataset online.

The expected sample was 8,000 to 10,000 responders across Canada – Canada’s population is 38.01 million (2020). Inclusion criteria: any person of age 18 years and older, with access to technology, and who were able to read/answer the questions in English or French. The enrolment period lasted from August 2020 through March 2021.

The study population was self-selected. Initially, for the first 4 months, we (research team members, neighbors, colleagues, and people from different volunteering associations) distributed the questionnaire to people from Eastern Ontario, using emails to personal connections within the region (aiming to first obtain a description of the phenomena in this part of the country). We obtained 405 answers, from the South East Ontario Region’s population of 1.76 million (which includes the cities of Ottawa, Brockville, Cornwall, Kingston and Pembroke, and towns of Gananoque, Prescott and Smith Falls, and counties of Prescott and Russell, Stormont, Dundas and Glengarry, Lanark, Leeds and Grenville, Frontenac and Lennox, and Addington). Subsequently, for the last four distribution months, we circulated the questionnaire using the most common media tools (Facebook, LinkedIn, Research Gate, and Twitter). Unfortunately, the rate of response fell below expectations. This was likely due in part to the important burden imposed by the pandemic, with people using media channels to comfort themselves rather than further focusing on worries and uncertainty. The increased availability of people contacted directly could be explained by a sense of responsibility and reciprocity, compared to the wide media approach which allows for anonymity and the freedom to postpone the engagement. Some responders provided us with feedback; from this, we learned that the 107 answers from the Canada-wide distribution came mainly from Nova Scotia, British Columbia, Québec, and Alberta.

Of the 512 total responders, we excluded 4 (who were under the age of 18 years), making the working sample of 508 persons. Distress and clinical depression were identified with the use of a cut-off and a previously developed algorithm, respectively. According to a previously developed method by the Greek site (13, 14), both a cut-off score of 23/24 for the CES-D as well as a derived algorithm were used, with cases of major depression being those identified by both methods. Those identified by only one of these tools are considered cases of distress (false positive cases in terms of depression). The two bibliographical studies show that 10% of the population are falsely diagnosed with depression by CES-D; these respondents were not depressed but had high-stress levels. A combined approach with the algorithm plus the cut-off method was able to clarify if the person is depressed or stressed.

### Measures

The online questionnaire (QAIRE) registered demographic, health data, previous psychiatric history, current symptoms of anxiety [STAI-Y1 state, (15)], depression (CES-D), and suicidality (RASS), as well as changes in sleep, sexual activity, family relationships, finance, eating, exercising, and religion/spirituality. Each question of the QAIRE questionnaire protocol was given an ID code, reflecting the part of the protocol it belongs to, with a capital letter (defining 12 sections, coded from A to P) and a number (total 121 questions, with Likert scale response options) to denote its position within that protocol part. Throughout the results, these ID codes are used for increased accuracy (Supplementary Appendix 4).

### Statistical Analysis

A method of simplified post-stratification was used to create a standardized study sample with characteristics as close as possible to those of the general population. Considering the small sample size, we measured the Cronbach’s alpha for the scales we used: alpha CES-D - 0.92; alpha STAI-Y1-0.93; alpha RASS-0.42.

Descriptive statistics were calculated and descriptive tables were created for the variables under investigation. Chi-square tests were used for the comparison of frequencies when categorical variables were present, and for the *post hoc* analysis of the results, a Bonferroni-corrected method of pair-wise comparisons was used. We performed multiple forward stepwise linear regressions. Multifactorial Analysis of Variance (MANOVA) was used to test for the main effect as well as the interaction among categorical variables, with Tukey’s *post hoc* test being employed to investigate which variables could contribute to the development of others.

## Results

### Study Sample

Of the 508 responders, 72.2% were females aged 42.57 ± 14.00 years; 27.2% were males aged 42.24 ± 15.49 years; and 0.6% were others aged 46.33 ± 17.79 years (Table 1: Descriptive Data).

**TABLE 1:** Descriptive statistics of the original (raw) and the standardized study samples in comparison to the general population.

	General population aged 18–87 years old		Raw study sample		Standardized study sample
			
	N^1^	%	*N*	%	%
**Sex**					
Males	9.98 mil	48.49	138	27.2	48.5
Females	10.6 mil	48.94	367	72.2	51.5
Other	0.75 mil	0.34	3	0.6	0.3
**Age**					
Males mean age	40.1		43.01		43.01
Females mean age	42.2		42.57		42.57
Other mean age			46.33		
**Work status**					
Total population	20.58 mil		508	100	100
Unemployed	1.89 mil		17	3.4	4.3
Students	1.78 mil		56	11	12.3
Self-employed	2.9 mil		134	26.3	27.7
Civil servant	3.6 mil		230	45.3	42
Disability pension	6.2 mil		4	0.8	1.2
Other			28	5.5	5.0
**Family and household**					
Married	14,547,623		305	60	59.5
Lives alone	3,969,790		75	14.8	14.7
2-people household	4,834,605		202	39.8	37.2
3-people household	2,140,640		85	16.7	17.6
4-people household	1,946,275		106	20.9	22.0
>5 people household	1,180,770		40	7.9	8.5
**Mental health history**					
History of deliberate harm			81	15.9	
History of suicide attempt			30	5.9	

*^1^Source: www.statista.com.*

### Living Situation, Family Status, and Relationships

Only 18.3% of the study sample was living in the country’s capital (Ottawa), 15.2% in a city of more than one million inhabitants, and 42.9% in a city with 100,000 to 1 million inhabitants. More than half (65%) of responders were married or common-law partners (marital status); only 14.6% were living alone (with nobody else under the same roof); and 45.9% had three or more people living in the house during the lockdown. In our sample, 243 responders (47.8%) had no children at all, while 28.1% have two children. For families with children, 22.7% stated that it was more difficult to manage their daily life and behavior than before. The responders stated that they were a close relative or caretaker of a person that belongs to a vulnerable group in 45.5% of cases. During the lockdown, the family relationships became more conflictual in 23.8% of cases, less conflictual in 20.3% of cases, and 55.9% of responders reported no changes. The overall quality of relationships with other family members was reported to have improved for 27.9% of responders, worsened for 18.8% of responders, and remained unchanged for 58.5%. The basic daily routine (waking up in the morning, regular meals and sleeping hours, and activities) was affected in 91.3% of cases, with 64.4% of responders noting this to be the case either “most of the time” or “always.”

### Education, Work, and Finances

A 88.8% of responders had a bachelor’s degree or higher. In terms of employment, 45.3% were civil servants, approximately 17% were working in the private sector, 11% were college or university students, and 10.7% were retired or were not working for a variety of reasons. From our sample, 29.9% were working in the healthcare sector (6.5% were doctors, 5.1% were nurses, 3.1% were administrative staff in hospitals, and 15.2% other healthcare professions/hospital staff). The percentage of people that did not work during the lockdown was 15.2%. Changes in finances due to the outbreak were reported as worse in 28.4% of cases, better in 21.6%, and unchanged in 50%.

### General Somatic Health and Lifestyle Changes

A history of chronic somatic diseases, such as asthma, diabetes mellitus, and hypertension was reported by 28.5% of responders. A worsening sleep pattern was reported by 47% of responders; 55.1% tended to stay up late at night and slept for many hours during the day; 37.4% reported experiencing dreams in which they felt trapped; 8% were taking sleeping pills for insomnia.

And 84.4% of responders acquired an internet-related habit they didn’t have before (e.g., a new FB account, engaging in cybersex, or gambling); a majority of respondents (80.7%) observed that the internet took up more of their time than usual.

Changes in their sexual life were reported by 37.8% of responders, with a decrease in desire for sexual intercourse reported in 20.9% of cases, and 20.7% of responders characterizing the frequency of their sexual intercourse as clearly inadequate. However, 30.5% believed that sex helps in dealing with stress and anxiety either “much” or “very much,” while 25% did not believe it does so at all.

Physical activity was not affected by lookdown for 20.9% of responders, while it decreased by 44.3% and increased by 34.9%. A great number of subjects (90%) stated that exercise helps with anxiety prevention, with 49.5% saying it does so either “much” or “very much.”

Eating habits were unchanged for 39.0% of responders, 41.7% were eating larger amounts of food or more meals per day than usual, and 30.5% reported eating healthier than usual. An increase in body weight was reported by 43% of responders.

During the lockdown, there were reported positive changes (using less) in smoking (11.6%), drinking patterns (15.2%), and illegal substance use (11.2%). A worsening use pattern was reported by 9.3% of responders for smoking, 29.3% for drinking (more than one drink or its equivalent every day), and 4.1% for illegal drugs.

Changes in religious/spiritual beliefs were present in 34% of those surveyed.

### Conspiracy Theories

Supplementary Appendix 3 summarizes the responses to all conspiracy theories, by current clinical depression and history of any mental disorder. We did not find a significant correlation between any theory and current depression and/or history of mental illness.

### Mental Health Data

#### Emotional Status

Increased anxiety during the lockdown was reported by 69.3% of those surveyed, and more than 18% reported that it increased “much” (24.8% reported unchanged and 5.7% reported decreased anxiety during the lookdown). The rate of suicidal thoughts increased for 19.5% of responders during the lockdown.

Depression at the time of completing the survey was reported by 22% (112 individuals), while distress was present in 18.4% of responders.

The subjective (by answers to specific questions) and objective (CES-D) emotional state is represented in Table 2.

**TABLE 2:** Emotional state by answers to specific questions (subjective) and clinical depression considering both CES-D methods (objective).

Question	Scoring	%
F21. How much has your emotional state changed in relation to the appearance of anxiety and insecurity compared to before the COVID-19 epidemic?	It got a lot worse	18.3
	It got a little worse	51.0
	Neither better nor worse	24.8
	It’s a little improved	4.1
	It has improved a lot	1.8
G21. How much has your emotional state related to the experience of joy or melancholy changed in comparison to before the COVID-19 epidemic?	It got a lot worse	13
	It got a little worse	46.3
	Neither better nor worse	35.0
	It’s a little improved	4.3
	It has improved a lot	1.4
O11. How much has your tendency to think about death and/or suicide changed, compared to before the outbreak of COVID-19 epidemic?	Very much increased	3.5
	Increased a bit	15.9
	Neither increased nor decreased	75.8
	Decreased a bit	3.7
	Very much decreased	1.0
Clinical depression according to both CES-D methods	No depression	59.6
	Depression according only to CES-D cut off^1^	40.2
	Depression according only to CES-D algorithm^1^	22.2
	Depression according to both methods^2^	22.0

*^1^Distress. ^2^Clinical depression.*

The rate of clinical major depression and distress was higher in females (Table 3).

**TABLE 3:** Rate of distress and depression by gender.

	No symptoms (*n* = 303)	Distressed (*n* = 93)	Depressed (*n* = 112)
**Sex (% of group)**			
Males	100 (33.0)	16 (17.2)	22 (19.6)
Females	201 (66.3)	77 (82.8)	89 (79.5)
Other	2 (0.7)	0	1 (0.9)
**Mean Age (SD)**	44.4 (14.3)	38.5 (14.6)	41.6 (13.2)

The health care workers, working on the front line during the pandemic, were the category of people most exposed to contracting the virus. The rate of distress versus major depression among health care professionals was not as high as expected (Table 4).

**TABLE 4:** Rate of distress and depression by employment in health care sector.

Employment in health sector (% of group)	No symptoms (*n* = 303)	Distressed	Depressed (*n* = 112)
Doctor	20 (6.6)	9 (9.7)	4 (3.6)
Nurse	18 (5.9)	2 (2.2)	6 (5.4)
Other clinicians	41 (13.5)	11 (11.8)	18 (16.1)
Administrative staff	9 (3.0)	5 (5.4)	2 (1.8)
Other hospital staff	5 (1.7)	1 (1.1)	1 (0.9)
Does not work in health sector	210 (69.3)	65 (69.9)	81 (72.3)

#### Relationship Between Depression and Previous History of Psychiatric and/or Chronic Somatic Disorders (Chi-Square Tests)

More than half of the responders (52.1%) reported having no history of mental disorders. A history of anxiety was present in 16% of responders, bipolar disorders in 1.2%, and psychosis in 0.4%, while a history of depression was present in 26.4%.

The relationship between depression and previous history of psychiatric and/or chronic somatic disorders (Chi-square tests) is represented in Table 5.

**TABLE 5:** The relationship between depression and previous history of psychiatric and/or chronic somatic disorders (Chi-square tests).

History	Control	Distressed	Depressed	% Depressed	Chi-square (*df* = 2)
Any mental disorder	**107a (143.1)**	52a (43.9)	**81a (52.9)**	33.8%	48.37, *p* < 0.001
No mental disorder	**196b (159.9)**	41a (49.1)	**31b (59.1)**	11.6%	
Anxiety disorder	48a (48.9)	18a (15.0)	16a (18.1)	19.5%	1.02, *p* = 0.602
No anxiety disorder	255a (254.1)	75a (78.0)	96a (93.9)	22.5%	
Depressive disorder	**49a (80.5)**	29a (24.7)	**57a (29.8)**	42.2%	51.76, *p* < 0.001
No depressive disorder	**254b (222.5)**	64a (68.3)	**55b (82.2)**	14.7%	
Other disorder	10a (13.7)	5a (4.2)	8a (5.1)	34.8%	2.98, *p* = 0.225
No other disorder	293a (289.3)	88a (88.8)	104a (106.9)	21.4%	
Self-harm	**31a (48.3)**	16a (14.8)	**34a (17.9)**	42.0%	24.85, *p* < 0.001
No self-harm	**272b (254.7)**	77a (78.2)	**78b (94.1)**	18.3%	
Suicide attempt	**12a (17.9)**	5a (5.5)	**13a (6.6)**	43.3%	8.66, *p* = 0.013
No suicide attempt	**291b (285.1)**	88a (87.5)	**99b (105.4)**	20.7%	
Chronic somatic condition	83a (86.5)	24a (26.5)	38a (33.9)	26.2%	2.13, *p* = 0.345
No chronic somatic condition	220a (216.5)	69a (66.5)	74a (80.0)	20.4%	

*The bold values are statistically significant.*

The highest Relative Risk (RR) to develop depression concerned the coexistence of history of depression and self-harm/attempts (RR = 4.71) (Table 6).

**TABLE 6:** The relative risk to develop depression in people with history of various mental disorders versus participants with no mental health history.

	Without history of self-harm or suicide attempt		With history of self-harm or suicide attempt	
		
History	%	RR	%	RR
No history of mental disorder	10.2	1.00	41.7	4.09
Any mental disorder	30.5	2.99	41.1	4.03
Anxiety disorder	17.9	1.75	26.7	2.62
Depressive disorder	38.8	3.80	48.0	4.71
Other disorders (e.g., psychosis, bipolar)	40.0	3.92	25.0	2.45

#### Prediction of Changes in the Mental State During the Pandemic (Forward Stepwise Multiple Linear Regression)

Dependent variables: Change in anxiety, change in depressive affect, presence of distress or depression, change in suicidal thoughts; 54 Independent variables (Supplementary Appendix 1). In Supplementary Appendix 2, we illustrated the protective factors (in green) and the risk factors (in red) for each of the four dependent variables (change in anxiety, change in depressive affect, presence of distress or depression, and change in suicidal thoughts). In our analysis, we included an entity named CORE factors, that were consistent across all four of the psychopathology variables; two of them acted as protective factors (keeping a basic routine during the lockdown and the improved sleep) and two as risk factors (gender-female and conflicts with family). The other factors included specifics in the equation of each of the four changes in mental status discussed (Supplementary Appendix 3).

## Discussion

This study aimed to investigate the rate of clinical depression in the adult population during the COVID-19 pandemic, as well as changes in anxiety, distress, suicidal ideation, and their relations with several personal and interpersonal/social variables. We found that the rate of suicidal thoughts increased for 19.5% of responders during the lockdown. Depression was reported in 22% of participants, while distress was present in 18.4%.

### Our Sample

Comprised 508 subjects, with a better representation for individuals living in Eastern Ontario, than Canada wide. However, the standardized study sample, from Table 1, provided close values. We used these values (Table 1) only to ensure that the demographic composition of our sample is comparable to the general population (16) – in our case, the Canadian population. Our results reflect the raw data and not the standardized sample. Through our analysis, we followed the same steps and strategies used in the parent study COMET –G. Through our analysis, we followed the same steps and strategies used in the parent study COMET –G. Gender/age distribution showed 72.2% females aged 42.57 ± 14.00 years; 27.2% males aged 42.24 ± 15.49 years; and 0.6% others aged 46.33 ± 17.79 years.

### Living Situation, Family Status, and Relationships

A significant percentage of responders reported deterioration in family dynamics during the lockdown (conflicts, change for worse in the quality of relationships). As expected, changes in basic routine affected 91.3% of subjects, with routine being changed “most of the time” or “always” for 64.4% of responders. Fountoulakis found that a higher number of individuals in Greece were able to maintain their daily routine (18.13%) during the lockdown.

The families with children (52.8% in our sample) indicated struggling to manage their behaviors more than before the pandemic in 22.7% of responses, similar to Greek families (27.43%). In Italy (17), research conducted on 1226 parents found that 17% of their sample experienced significant parenting-related exhaustion, with mothers more severely affected. Greater parenting-related exhaustion was predicted by psychological distress, lower parental resilience, motherhood, fewer perceived social connections, and being single, as well as having a child with special needs, having a large number of children, and having younger children.

The rate of people being either relatives or caretakers of vulnerable persons was slightly higher in our sample (45.5%) than the one communicated by the COMET-G Study (44.41%).

Under 15% of responders from our sample were living alone (still higher than the responses communicated by the COMET-G Study, of around 10% worldwide), becoming even more isolated during the pandemic. In addition, fewer than 20% of our subjects reported a decrease in communication with the extended family.

### Education, Work, and Finances

Our sample was comprised of a high number of highly educated people with more than bachelor’s degree as their educational status (88.8%), higher than the general Canadian population where only 64% have higher education. This is probably due to the self-selection of responders. The worldwide percentage of people with higher education is 75%, according to the COMET-G Study [COMET-G, (18)]. In terms of employment, 11% were college or university students, 10.7% were retired or were not working for a variety of reasons, and almost 30% were working in the health sector. A higher number of people were working during the pandemic in our sample than worldwide (78.4% compared with 66.14%). It would be interesting to know the percentage of people working remotely, from home (QAIRE had no question targeting this aspect). There are differences in changes in mental health during the lockdown, modulated by the type of work [see section “Relationship Between Depression and Previous History of Psychiatric and/or Chronic Somatic Disorders (Chi-Square Tests)”]. Despite the increased number of people not working during the lockdown, 21.6% of responders from our sample stated that their finances have improved, while 50% stated that their financial status was unchanged. This is one of the protective factors for anxiety and depression and could be explained by the generous compensatory financial support through Canada’s COVID-19 Economic Response Plan; for example, CERB (Canada Emergency Response Benefit), provided as financial support to employed and self-employed Canadians who were directly affected by COVID-19, as well as Canada Emergency Student Benefit (CESB), and Support for vulnerable people (homeless, indigenous communities, senior). Another explanation for this preservation in financial status is the abrupt changes in lifestyle which may have reduced spending (beauty services, restaurants, and shopping malls closed; inability to travel or pursue other hobbies such as golfing, skiing; theatres and arenas closed, etc.). And finally, we suspect that the financial status was preserved by a lower percentage of people without work (21.6%) compared with the global general population of 33.86% [COMET-G, (18)]; in Greece, this percentage was 47.37% (19).

An interesting survey conducted in Korea in December 2020 found that among the 322 participants, the prevalence of probable depression and GAD were 19.3 and 14.9%, respectively, with high rates of probable depression (23.3%) and GAD (19.4%) among persons currently having job-related and financial issues. Decreased access to nature/greenspaces during the lockdown were significantly associated with depression; an alternative explanation was that those experiencing poor mental health may be less likely to visit green spaces during the pandemic (20).

### General Somatic Health and Lifestyle Changes

Are extremely important in anxiety determinism (worsening of sleeping pattern, eating more, drinking more, increase in body weight, increased time spent on the internet). A worsening sleep pattern was reported by almost half of our sample, with more than half (55.1%) tending to stay up late at night, which could be partially explained by the increased interest in internet use. An important percentage of people (84.4%) acquired a new internet-related habit during the pandemic (e.g., a new FB account, engaging in cybersex, or gambling), while the majority (80.7%) observed that the internet took up more of their time than usual. A study of the interactions between anxiety levels and life habit changes in the Russian general population during the pandemic lockdown (21) concluded that factors of decreased physical activity and sleep disturbances related to the lockdown, as well as excessive internet browsing for information about COVID-19, emerged as risk factors for increased anxiety, more notably in women than in men. The decreased physical activity in our sample was reported by 44.3% of responders and close to the 45.05% reported worldwide [COMET-G, (18)]. The decrease in smoking and use of illegal drugs was seen equally in Canadians as in the COMET-G Study, and both studies found comparable changes related to sexual life, eating, and sleep patterns. However, 34% of interviewed people in Canada increased their religious beliefs, compared to the 19.18% communicated in the COMET-G Study. This could be considered a protective factor for suicidal attempts, if not also for distress.

### Conspiracy Theories

In the COMET-G Study, Fountoulakis (19) observed that some conspiracy theories are exerting a protecting effect at certain phases. We did not find a significant correlation between any theory and current depression and/or history of mental illness.

For Canadians, out of the seven theories inquired about, only the belief that “COVID-19 appeared accidentally from human contact with animals” was embraced by almost 90%, with 53.5% believing “much” and “very much” in this.

### Changes in Mental Health

#### Emotional Status During the Pandemic

Increased anxiety during the lockdown was reported by 69.3% in our sample. Major depression was detected in 22%, while distress was present in 18.4%. When compared with the worldwide rate of 17.80% with major depression and 16.71% with distress, calculated under the same circumstances and time [COMET-G, (18)], there was a higher rate of both in Canada. A low rate of increased anxiety during the pandemic was reported in Pakistan, and the authors concluded that it “demonstrates either the resilience of Pakistanis or the lack of understanding of the seriousness of the situation” (22). Higher distress levels were reported by Yael (23), who imagined the profile of individuals with elevated distress as: “being younger, female, not in a relationship, having a below-average income, being diagnosed with the disease, living alone during the outbreak, having a close other in a high-risk group, and negatively self-rating one’s health status.” Chang et al. (24) found that fear of COVID-19 among people with mental illness was associated with psychological distress (including depression, anxiety, and stress), while the present study found that mental disorder is associated with depression only.

The rate of suicidal thoughts increased for 19.5% of responders during the lockdown, while the COMET-G study found an increase of only 17.16%. There is a large heterogeneity among countries in the description of suicidal behavior during the pandemic, again, possibly due to the different times of rating and different instruments used (18, 25, 26).

#### The History of Any Mental Disorder

A history of self-harm and suicidality represented a risk factor for developing depression. People with a history of any mental disorder had higher rates of developing depression than people with no such history; these rates are higher for Canadians when compared with the global population as reported in Fountoulakis’ paper (32% vs. 13.07%). In Fountoulakis’ paper, the highest risk was associated with a history of self-harm/suicidality/bipolar disorder (RR 5.88), while in the Canadian population, the higher risk was represented by a history of self-harm/suicidality/depression (RR 4.80). People with no history of mental illness had a lower risk of developing depression (RR 1.00), the same risk for Canadians as Fountoulakis’ general population. The presence of a chronic somatic condition was not a significant risk factor for the development of depression in Canadians, compared with Fountoulakis’ general population where the RR was 1.22.

A history of self-harm or suicidality emerged as a risk factor even for persons without a reported mental health history, of which 41.67% develop depression in the presence of this risk factor. The combination of both self-harm and a history of suicidal attempts with specific mental health history revealed that subjects without any such history had the lowest rate of current depression (10.00%), while the presence of previous self-harm/attempts increased the risk in subjects with past anxiety (26.67%), depression (48.00%), and other mental disorder (25.00%).

#### Prediction of Changes in the Mental State During the Pandemic (Forward Stepwise Multiple Linear Regression)

Of the protective and risk factors modulating the change in anxiety, the change in depressive affect, the presence of distress or depression, and the change in suicidal thoughts, we found four factors (CORE) that were consistent across all four of the psychopathology variables. Two of them acted as protective factors (keeping a basic routine during the lockdown and the improved sleep) and two as risk factors (gender-female and conflicts with family). A systematic review conducted on PubMed, Embase, Medline, Web of Science, and Scopus in 2020 [Jiaqi (27)] showed relatively high rates of symptoms of anxiety (6.33 to 50.9%), depression (14.6 to 48.3%), and psychological distress (34.43 to 38%) in the general population during the COVID-19 pandemic in China, Spain, Italy, Iran, the United States, Turkey, Nepal, and Denmark. Risk factors associated with distress measures included female gender, younger age group (≤40 years), presence of chronic/psychiatric illnesses, unemployment, student status, and frequent exposure to social media/news concerning COVID-19.

## Conclusion

In our sample, we found a greater prevalence of depression but not distress in individuals with a history of any mental disorder. Based on the multiple regression analysis, we found four CORE factors equally influencing all considered changes in mental health during the lockdown: gender, quality of sleep, daily routine, and conflicts with family.

In the Canadian population, two major changes acted as protective factors, significantly expressed when compared with the worldwide tendencies: the lesser financial difficulties (support offered by the Government, higher number of subjects working, even if from home) and an increase in religious beliefs. The impact was not on the general rate of major depression, distress, and suicidal ideation (these were higher in Canadians than worldwide), but on the lower relative risk to develop depression in the presence of a history of mental disorders.

Almost 90% of Canadians believed in the most probable real story of COVID source of provenience.

Our research findings will help better understand the factors involved in the determinism of depression, suicidality, and distress in the Canadian population during critical situations. These could be taken into consideration when organizing future mental health programs and interventions, aiming to protect at-risk populations.

### Strengths and Limitations

The strength of the current paper derives from the large bulk of information obtained, which allowed us to have an idea of how the pandemic affected Canadians’ life.

The limitations derive from the small sample size and the method in which data were collected (anonymously online through responder self-selection). The changes during the lockdown discussed here are only perceived changes as we do not have a pre-lockdown measure. The low internal consistency of the RASS (0.42) in the present study is another limitation.

## Data Availability Statement

The original contributions presented in the study are included in the article/Supplementary Material, further inquiries can be directed to the corresponding author.

## Ethics Statement

The studies involving human participants were reviewed and approved by the Health Science Research Ethics Board at Queen’s University, Kingston, ON, Canada. Written informed consent for participation was not required for this study in accordance with the national legislation and the institutional requirements. Written informed consent was obtained from the individual(s), and minor(s)’ legal guardian/next of kin, for the publication of any potentially identifiable images or data included in this article.

## Author Contributions

All authors listed have made a substantial, direct, and intellectual contribution to the work, and approved it for publication.

## Conflict of Interest

The authors declare that the research was conducted in the absence of any commercial or financial relationships that could be construed as a potential conflict of interest.

## Publisher’s Note

All claims expressed in this article are solely those of the authors and do not necessarily represent those of their affiliated organizations, or those of the publisher, the editors and the reviewers. Any product that may be evaluated in this article, or claim that may be made by its manufacturer, is not guaranteed or endorsed by the publisher.
