# Supplementary material for: COVID-19 Pandemic: The Impact of COVID-19 on Mental Health and Life Habits in the Canadian Population
Source: Front Psychiatry. 2022 Jun 29;13:871119. doi: 10.3389/fpsyt.2022.871119 (PMC9295836; doi:10.3389/fpsyt.2022.871119)
Supplement: Supplementary file 1 [file Table_1.DOCX]

|  | **Change in anxiety (F21)**  **R² =** **.377; F(18, 486) = 16.37  *p* < .001; SE of est: 0.682** | | | | | **Change in depressive affect (G21)**  **R² = .393; F(12, 492) = 26.52  *p* < .001; SE of est: 0.641** | | | | | **Development of dysphoria or depression**  **R² = .475.; F(17, 487) = 25.88  *p* < .001; SE of est: 0.606** | | | | | **Change in suicidal thoughts (O11)**  **R² = .148; F(10, 494) = 26.21 *p* < .001; SE of est: 0.552** | | | | |
| --- | --- | --- | --- | --- | --- | --- | --- | --- | --- | --- | --- | --- | --- | --- | --- | --- | --- | --- | --- | --- |
|  | **B** | **SE** | **β** | **t** | **p** | **b** | **SE** | **β** | **t** | **p** | **b** | **SE** | **β** | **t** | **p** | **b** | **SE** | **β** | **t** | **p** |
| Intercept | -0.34 | 0.16 |  | -2.20 | .028 | -0.52 | 0.16 |  | -3.22 | .001 | 0.49 | 0.17 |  | 2.89 | .004 | -0.10 | 0.14 |  | -0.74 | .459 |
| **Demographics** |  |  |  |  |  |  |  |  |  |  |  |  |  |  |  |  |  |  |  |  |
| Sex (A1) - ‘other’ was not included |  |  |  |  |  |  |  |  |  |  |  |  |  |  |  |  |  |  |  |  |
| Age (A2) |  |  |  |  |  |  |  |  |  |  |  |  |  |  |  |  |  |  |  |  |
| Number of persons in household (A5) |  |  |  |  |  |  |  |  |  |  | -0.07 | 0.02 | -0.10 | -2.87 | .004 |  |  |  |  |  |
| Number of children (A6) |  |  |  |  |  |  |  |  |  |  |  |  |  |  |  |  |  |  |  |  |
| Education level (A7) |  |  |  |  |  |  |  |  |  |  |  |  |  |  |  |  |  |  |  |  |
| **Work and finance** |  |  |  |  |  |  |  |  |  |  |  |  |  |  |  |  |  |  |  |  |
| Employment (A9) |  |  |  |  |  |  |  |  |  |  |  |  |  |  |  |  |  |  |  |  |
| Working in health sector (A10) | -0.16 | 0.07 | -0.09 | -2.34 | .020 |  |  |  |  |  |  |  |  |  |  |  |  |  |  |  |
| Continue to work during lockdown (A11) |  |  |  |  |  |  |  |  |  |  |  |  |  |  |  |  |  |  |  |  |
| Change in economic situation (E7) | 0.09 | 0.04 | 0.09 | 2.37 | .020 |  |  |  |  |  | -0.07 | 0.03 | -0.08 | -2.29 | .022 |  |  |  |  |  |
| **Health** |  |  |  |  |  |  |  |  |  |  |  |  |  |  |  |  |  |  |  |  |
| Condition of general health (B1) | 0.06 | 0.03 | 0.08 | 2.04 | .042 | 0.13 | 0.03 | 0.18 | 4.68 | < .001 | -0.08 | 0.03 | -0.11 | -3.07 | .002 |  |  |  |  |  |
| Presence of a chronic medical condition (B2) |  |  |  |  |  |  |  |  |  |  |  |  |  |  |  |  |  |  |  |  |
| Change in appetite (I1) |  |  |  |  |  |  |  |  |  |  | -0.08 | 0.03 | -0.10 | -2.79 | .006 |  |  |  |  |  |
| Change in nutrition (I2) | 0.11 | 0.05 | 0.09 | 2.44 | .015 | 0.10 | 0.04 | 0.09 | 2.36 | .019 | -0.10 | 0.04 | -0.08 | -2.28 | .023 |  |  |  |  |  |
| Change in body weight (I3) |  |  |  |  |  |  |  |  |  |  |  |  |  |  |  |  |  |  |  |  |
| Change in quality of sleep (L1) | 0.16 | 0.04 | 0.15 | 3.68 | < .001 | 0.19 | 0.04 | 0.19 | 4.86 | < .001 | -0.12 | 0.04 | -0.12 | -3.08 | .002 | 0.06 | 0.03 | 0.08 | 1.70 | .090 |
| Staying up late and sleeping in (L2) | 0.13 | 0.03 | 0.17 | 3.84 | < .001 |  |  |  |  |  |  |  |  |  |  |  |  |  |  |  |
| Takes sleeping pills at night (L3) |  |  |  |  |  |  |  |  |  |  |  |  |  |  |  | -0.06 | 0.02 | -0.11 | -2.64 | .009 |
| Dreams of feeling trapped (L4) | -0.09 | 0.03 | -0.11 | -2.66 | .008 | -0.12 | 0.03 | -0.16 | -3.91 | < .001 | 0.08 | 0.03 | 0.10 | 2.62 | .009 |  |  |  |  |  |
| Smoking before pandemic (M1) |  |  |  |  |  |  |  |  |  |  |  |  |  |  |  |  |  |  |  |  |
| Alcohol use before pandemic (M2) |  |  |  |  |  |  |  |  |  |  |  |  |  |  |  |  |  |  |  |  |
| Use of illicit substances before pandemic (M3) |  |  |  |  |  |  |  |  |  |  |  |  |  |  |  |  |  |  |  |  |
| Change in smoking (M4) |  |  |  |  |  |  |  |  |  |  |  |  |  |  |  |  |  |  |  |  |
| Change in drinking (M5) |  |  |  |  |  |  |  |  |  |  |  |  |  |  |  |  |  |  |  |  |
| Change in illicit substance use (M6) |  |  |  |  |  |  |  |  |  |  |  |  |  |  |  |  |  |  |  |  |
| **Family/social** |  |  |  |  |  |  |  |  |  |  |  |  |  |  |  |  |  |  |  |  |
| Being a caregiver of a person belonging to a vulnerable group (B4) |  |  |  |  |  |  |  |  |  |  |  |  |  |  |  |  |  |  |  |  |
| Need to communicate with family (E1) |  |  |  |  |  |  |  |  |  |  |  |  |  |  |  |  |  |  |  |  |
| Desire to receive emotional support from family (E2) | -0.16 | 0.04 | -0.15 | -3.98 | < .001 | -0.13 | 0.04 | -0.14 | -3.61 | < .001 | 0.11 | 0.04 | 0.11 | 3.09 | .002 |  |  |  |  |  |
| Conflicts within family (E3) | -0.11 | 0.04 | -0.12 | -3.16 | .002 | -0.11 | 0.03 | -0.13 | -3.48 | .001 | 0.08 | 0.03 | 0.09 | 2.49 | .013 | -0.08 | 0.03 | -0.12 | -2.77 | .006 |
| Change in quality of relationships within family (E4) |  |  |  |  |  |  |  |  |  |  |  |  |  |  |  |  |  |  |  |  |
| Keeping a basic routine during lockdown (E5) | 0.09 | 0.04 | 0.09 | 2.11 | .035 | 0.10 | 0.04 | 0.10 | 2.68 | .008 | -0.14 | 0.04 | -0.15 | -4.07 | < .001 | 0.07 | 0.03 | 0.10 | 2.38 | .020 |
| Difficulty managing children (E6) |  |  |  |  |  |  |  |  |  |  |  |  |  |  |  |  |  |  |  |  |
| Satisfaction with frequency of sex (N1) | 0.07 | 0.03 | 0.10 | 2.56 | .011 | 0.07 | 0.03 | 0.09 | 2.61 | .009 | -0.06 | 0.02 | -0.08 | -2.32 | .021 |  |  |  |  |  |
| Change in desire for sex (N2) |  |  |  |  |  |  |  |  |  |  |  |  |  |  |  |  |  |  |  |  |
| Satisfaction with sex life (N3) |  |  |  |  |  |  |  |  |  |  |  |  |  |  |  |  |  |  |  |  |
| Use of sex to cope with stress (N4) | -0.07 | 0.02 | -0.11 | -2.90 | .004 | -0.07 | 0.02 | -0.11 | -3.11 | .002 |  |  |  |  |  |  |  |  |  |  |
| Changes in religiousness/spirituality (P1) |  |  |  |  |  |  |  |  |  |  |  |  |  |  |  |  |  |  |  |  |
| **Mental health history** |  |  |  |  |  |  |  |  |  |  |  |  |  |  |  |  |  |  |  |  |
| History of anxiety (B5) |  |  |  |  |  |  |  |  |  |  |  |  |  |  |  |  |  |  |  |  |
| History of depression (B5) | -0.17 | 0.07 | -0.09 | -2.36 | .019 |  |  |  |  |  | 0.31 | 0.07 | 0.17 | 4.53 | < .001 |  |  |  |  |  |
| History of other mental disorder (B5) |  |  |  |  |  |  |  |  |  |  |  |  |  |  |  | -0.26 | 0.12 | -0.09 | -2.20 | .029 |
| History of self-harm/attempt (O12 and O13) |  |  |  |  |  |  |  |  |  |  | 0.16 | 0.08 | 0.07 | 2.04 | .041 |  |  |  |  |  |
| **The effect of the pandemic** |  |  |  |  |  |  |  |  |  |  |  |  |  |  |  |  |  |  |  |  |
| Fears of getting COVID-19 (C1) | -0.08 | 0.04 | -0.11 | -2.29 | .023 |  |  |  |  |  |  |  |  |  |  |  |  |  |  |  |
| Fears that a member of the family will get COVID-19 and die (C3) | -0.07 | 0.03 | -0.10 | -2.11 | .035 | -0.08 | 0.03 | -0.13 | -3.28 | .001 | 0.06 | 0.03 | 0.08 | 2.23 | .026 |  |  |  |  |  |
| Fear of being treated differently for getting COVID-19 (C4) |  |  |  |  |  |  |  |  |  |  | 0.06 | 0.03 | 0.09 | 2.31 | .021 |  |  |  |  |  |
| Time spent outside of house during lockdown (D1) |  |  |  |  |  |  |  |  |  |  |  |  |  |  |  |  |  |  |  |  |
| Currently locked up in the house (D2) |  |  |  |  |  |  |  |  |  |  |  |  |  |  |  |  |  |  |  |  |
| Satisfaction by availability of information (D4) |  |  |  |  |  |  |  |  |  |  |  |  |  |  |  |  |  |  |  |  |
| **Thoughts about measures taken** |  |  |  |  |  |  |  |  |  |  |  |  |  |  |  |  |  |  |  |  |
| Believe precautions work (C2) |  |  |  |  |  |  |  |  |  |  |  |  |  |  |  | 0.27 | 0.08 | 0.15 | 3.39 | .001 |
| Belief that personally taken measures are adequate (D3) |  |  |  |  |  | -0.10 | 0.04 | -0.10 | -2.79 | .006 | 0.06 | 0.03 | 0.06 | 1.85 | .065 | -0.09 | 0.31 | -0.12 | -2.87 | .004 |
| **Physical activity** |  |  |  |  |  |  |  |  |  |  |  |  |  |  |  |  |  |  |  |  |
| Exercise preventing anxiety (H1) |  |  |  |  |  |  |  |  |  |  |  |  |  |  |  |  |  |  |  |  |
| Importance of exercise (H2) |  |  |  |  |  |  |  |  |  |  |  |  |  |  |  |  |  |  |  |  |
| Change in exercise frequency (H3) |  |  |  |  |  |  |  |  |  |  |  |  |  |  |  |  |  |  |  |  |
| Impact of pandemic on exercise (H4) |  |  |  |  |  |  |  |  |  |  |  |  |  |  |  |  |  |  |  |  |
| **Beliefs in conspiracy theories** |  |  |  |  |  |  |  |  |  |  |  |  |  |  |  |  |  |  |  |  |
| The vaccine was ready before the virus broke out and they conceal it (J1) | 0.10 | 0.05 | 0.08 | 2.02 | .044 |  |  |  |  |  |  |  |  |  |  |  |  |  |  |  |
| COVID-19 was created in a laboratory as a biochemical weapon (J2) |  |  |  |  |  |  |  |  |  |  |  |  |  |  |  | 0.07 | 0.03 | 0.11 | 2.43 | .016 |
| COVID-19 is the result of 5G technology antenna (J3) |  |  |  |  |  |  |  |  |  |  |  |  |  |  |  |  |  |  |  |  |
| COVID-19 appeared accidentally from human contact with animals (J4) |  |  |  |  |  | 0.06 | 0.02 | 0.10 | 2.75 | .006 |  |  |  |  |  |  |  |  |  |  |
| COVID-19 has much lower mortality rate but there is terror-inducing propaganda (J5) |  |  |  |  |  |  |  |  |  |  |  |  |  |  |  |  |  |  |  |  |
| COVID-19 is a creation of the world’s powerful leaders to create a global economic crisis (J6) | -0.14 | 0.05 | -0.12 | -3.00 | .003 |  |  |  |  |  |  |  |  |  |  |  |  |  |  |  |
| COVID-19 is a sign of divine power to destroy our planet (J7) |  |  |  |  |  |  |  |  |  |  |  |  |  |  |  | -0.11 | 0.04 | -0.12 | -2.81 | .005 |
| **Online activity** |  |  |  |  |  |  |  |  |  |  |  |  |  |  |  |  |  |  |  |  |
| COVID-19 information causes worry (K1) |  |  |  |  |  |  |  |  |  |  | 0.11 | 0.02 | 0.17 | 4.89 | < .001 |  |  |  |  |  |
| Belief that COVID-19 information from internet is misleading (K2) |  |  |  |  |  |  |  |  |  |  |  |  |  |  |  |  |  |  |  |  |
| Increased internet usage (K3) | -0.10 | 0.03 | -0.15 | -3.69 | < .001 |  |  |  |  |  |  |  |  |  |  | -0.05 | 0.02 | -0.10 | -2.22 | .027 |
| Change in social media use (K4) |  |  |  |  |  |  |  |  |  |  |  |  |  |  |  |  |  |  |  |  |
| Development of new internet-related habit (K5) |  |  |  |  |  |  |  |  |  |  |  |  |  |  |  |  |  |  |  |  |

**Appendix 1. . Prediction of changes in mental state during the pandemic (Forward Stepwise Multiple Linear Regressi**
