# Supplementary material for: COVID-19 Pandemic: The Impact of COVID-19 on Mental Health and Life Habits in the Canadian Population
Source: Front Psychiatry. 2022 Jun 29;13:871119. doi: 10.3389/fpsyt.2022.871119 (PMC9295836; doi:10.3389/fpsyt.2022.871119)
Supplement: Supplementary file 2 [file Table_2.DOCX]

|  | **Stress** | | **Depres- sion** | **Suicid-**  **ality** | **Core factor** |
| --- | --- | --- | --- | --- | --- |
|  | **Anxiety** | **Depressive affect/dysphoria** |  |  |  |
| **Demographics** | | | | |  |
| Gender (female) |  |  |  |  |  |
| Number of people in household |  |  | **P** |  |  |
| **Work and finance** | | | | |  |
| Working in health sector | **R** |  |  |  |  |
| Worsening economic situation | **R** |  | **R** |  |  |
| **Health** | | | | |  |
| Worsening general health | **R** | **R** | **R** |  |  |
| Increasing appetite |  |  | **P** |  |  |
| Healthier eating | **P** | **P** | **P** |  |  |
| Improving sleep | **P** | **P** | **P** | **P** | **X** |
| Staying up late and sleeping in | **P** |  |  |  |  |
| Taking pills to sleep at night |  |  |  | **R** |  |
| Having dreams of feeling trapped | **R** | **R** | **R** |  |  |
| **Family/social** | | | | |  |
| Desire to get more emotional support from family | **R** | **R** | **R** |  |  |
| Conflicts with family | **R** | **R** | **R** | **R** | **X** |
| Keeping a basic routine during lockdown | **P** | **P** | **P** | **P** | **X** |
| Satisfaction with frequency of sex | **P** | **P** | **P** |  |  |
| Use of sex to cope with stress | **R** | **R** |  |  |  |
| **Mental health history** | | | | |  |
| History of depressive disorder | **R** |  | **R** |  |  |
| History of other disorder (e.g., psychosis, bipolar, etc.) |  |  |  | **R** |  |
| History of self-harm or suicide attempt |  |  | **R** |  |  |
| **The effect of the pandemic** | | | | |  |
| Fear of getting COVID-19 | **R** |  |  |  |  |
| Fear that a family member will get COVID-19 and die | **R** | **R** | **R** |  |  |
| Fear of being treated differently for COVID-19 |  |  | **R** |  |  |
| **Thoughts about measures taken** | | | | |  |
| Belief that COVID-19 precautions work |  |  |  | **P** |  |
| Belief that personally taken measures are adequate |  | **R** | **R** | **R** |  |
| **Beliefs in conspiracy theories** | | | | |  |
| Belief that COVID-19 appeared accidentally from human contact with animals (non-conspiracy) |  | **P** |  |  |  |
| **‘Threatening’ conspiracy theories** | | | | |  |
| Belief that COVID-19 is a creation of the world’s powerful leaders to create a global economic crisis | **R** |  |  |  |  |
| Belief that COVID-19 was created in a laboratory as a biochemical weapon |  |  |  | **P** |  |
| **‘Reassuring’ conspiracy theories** | | | | |  |
| Belief that vaccine was ready before the virus broke out and it was concealed | **P** |  |  |  |  |
| Belief that COVID-19 is a sign of divine power to destroy our planet |  |  |  | **R** |  |
| **Online activity** | | | | |  |
| Being worried by online COVID-19 information |  |  | **R** |  |  |
| Increase in internet usage | **R** |  |  | **R** |  |

**Appendix 2: Summary of risk and protective factors foe mental health changes**
