# Supplementary material for: COVID-19 Pandemic: The Impact of COVID-19 on Mental Health and Life Habits in the Canadian Population
Source: Front Psychiatry. 2022 Jun 29;13:871119. doi: 10.3389/fpsyt.2022.871119 (PMC9295836; doi:10.3389/fpsyt.2022.871119)
Supplement: Supplementary file 3 [file Table_3.DOCX]

|  |  |  | **Reassuring conspiracy theories** | | | | | | | | **Threatening conspiracy theories** | | | | | | | | **Total** | | **No believing in conspiracy theories** | |
| --- | --- | --- | --- | --- | --- | --- | --- | --- | --- | --- | --- | --- | --- | --- | --- | --- | --- | --- | --- | --- | --- | --- |
| **Current**  **Clinical**  **Depression** | **History**  **of any Mental Disorder** | ***n*** | **J1** | | **J5** | | **J7** | | **J1+J5+J7** | | **J2** | | **J3** | | **J6** | | **J2+J3+J6** | | **J1+J2+J3 +J5+J6+J7** | | **J4** | |
|  |  |  | ***M*** | ***SD*** | ***M*** | ***SD*** | ***M*** | ***SD*** | ***M*** | ***SD*** | ***M*** | ***SD*** | ***M*** | ***SD*** | ***M*** | ***SD*** | ***M*** | ***SD*** | ***M*** | ***SD*** | ***M*** | ***SD*** |
| **No** | **Yes** | 159 | 0.18 | 0.55 | 0.61 | 1.02 | 0.17 | 0.57 | 0.96 | 1.55 | 0.36 | 0.78 | 0.08 | 0.38 | 0.26 | 0.67 | 0.70 | 1.41 | 1.66 | 2.59 | 2.65 | 1.34 |
| **No** | **No** | 237 | 0.22 | 0.69 | 0.76 | 1.13 | 0.16 | 0.54 | 1.14 | 1.73 | 0.45 | 0.93 | 0.15 | 0.55 | 0.28 | 0.73 | 0.88 | 1.75 | 2.02 | 3.13 | 2.53 | 1.28 |
| **Yes** | **Yes** | 81 | 0.28 | 0.86 | 0.49 | 0.81 | 0.30 | 0.91 | 1.07 | 1.86 | 0.52 | 1.11 | 0.20 | 0.66 | 0.31 | 0.75 | 1.02 | 2.16 | 2.10 | 3.83 | 2.48 | 1.23 |
| **Yes** | **No** | 31 | 0.52 | 1.00 | 1.03 | 1.35 | 0.42 | 0.99 | 1.97 | 2.69 | 1.00 | 1.37 | 0.35 | 0.99 | 0.77 | 1.33 | 2.13 | 3.22 | 4.10 | 5.63 | 2.10 | 1.40 |
| **All Grps** | | 508 | 0.24 | 0.71 | 0.69 | 1.07 | 0.20 | 0.66 | 1.12 | 1.79 | 0.47 | 0.96 | 0.15 | 0.57 | 0.31 | 0.77 | 0.92 | 1.87 | 2.05 | 3.37 | 2.53 | 1.30 |

**Appendix 3: Means of responses to all conspiracy theories by current clinical depression and history of any mental disorder**
