# Supplementary material for: COVID-19 Pandemic: The Impact of COVID-19 on Mental Health and Life Habits in the Canadian Population
Source: Front Psychiatry. 2022 Jun 29;13:871119. doi: 10.3389/fpsyt.2022.871119 (PMC9295836; doi:10.3389/fpsyt.2022.871119)
Supplement: Supplementary file 4 [file Table_4.DOCX]

**ESTIMATING THE EFFECTS OF COVID-19 OUTBREAK ON MENTAL HEALTH**

The following questionnaire is an initiative of the Mental Health Sector of the Scientific Researches Institute of the Pan-Hellenic Medical Association. It assesses how the COVID-19 pandemic, either in itself or as a result of the measures adopted to control the outbreak, has affected various aspects of the mental function, needs and behaviours of the general population in Canada.

No identifying information is required for your participation in this study. This is to ensure that your answers are as sincere and unconstrained as possible.

By participating in this study, you are contributing to the advancement of our knowledge regarding mental health, in particular regarding how periods of crises and mass disasters and threats affect mental health. There are no results or evaluation of any kind available at the end of the survey.

**The questionnaire is long and while you may find it tedious, a detailed record of the various aspects of the problem is the only way to identify the mental health issues that will need addressing in the future.**

There are no risks associated with your participation in this study. However, it requires a fair bit of your time as there are many questions which cover several different, but equally important, aspects of human behavior.

All of the data that we receive from you in this questionnaire are anonymous and strictly confidential. For the scientific analysis, the data will be processed without your name (which you are not required to provide at any stage of the study). The researchers involved in this study are the only people authorized to examine survey responses. The protection of your privacy is our duty.

If you have any questions or concerns at any time regarding your participation in this study, the manner in which the study is conducted, the risks involved or your rights, please contact Professor Fountoulakis Konstantinos via e-mail to fountoul@auth.gr (3^rd^ Department of Psychiatry, University Hospital AHEPA hospital, Aristotle University of Thessaloniki, Greece).

Canadian Principal Investigator is Professor Milev, Roumen from Queen’s University, Kingston, ON, e-mail: [roumen.milev@queensu.ca](mailto:roumen.milev@queensu.ca)

This study has been reviewed for ethical compliance by the Queen’s University Health Sciences and Affiliated Teaching Hospitals Research Ethics Board.

**Α. GENERAL DATA**

**Sex**

- Male
- Female
- Other/I do not wish to define

**Date of Birth written with 4 digits (e.g. 1982) ***

Please enter your date of birth in the form of 4 digits (e.g. 1981, 1968, 1993 etc.)

**Place of residence during this period**

- Capital City
- City > 1 million population
- City (100.000 – 1 million population)
- Town (20.000 – 100.000 inhabitants)
- Town (<20.000 inhabitants)
- Rural area - Village

**Marital Status**

- Single
- Married (or in a civil partnership)
- Divorced (or estranged)
- Live with someone without an official relationship
- Widower
- Other

**How many people reside in the house you are staying in during this period (including yourself)?**

- 1 (I live alone)
- 2 people
- 3 people
- 4 people
- 5 or more people

**How many children do you have (regardless of whether they live in the same house with you or not)?**

- 0 (I do not have any children)
- 1 child
- 2 children
- 3 children
- 4 or more children

**Education**

- Elementary school or less (no more than 9 years of education)
- High school degree or equivalent (no more than 12 years of education)
- Bachelor’s degree
- Master’s degree
- Doctorate (PhD)

**Employment**

- Working at the Public Sector
- Salaried employee at the Private Sector
- Self-employed**/**Freelancer
- Retired
- Unemployed
- Housekeeping
- Retirement for health reasons
- Allowance for health reasons
- Not working by choice (e.g. living via corporate earnings etc.)
- University or College Student
- Other

**Employment in the Health Sector**

- I do not work in the Heath Sector
- Doctor
- Nurse
- Other healthcare profession with direct contacts with areas of clinical work
- Administrative employee at a hospital
- Other hospital staff

**Do you continue to exercise your profession during the period of the lockdown?**

- Yes
- No

**B. GENERAL STATE OF HEALTH AND COVID-19**

**In general, your health over the last month can be described as:**

- Excellent
- Very good
- Good
- Moderate
- Bad

**Do you suffer from any chronic medical condition (for example: diabetes mellitus, hypertension, asthma, etc.)?**

- Yes
- No

**If yes, please define your chronic medical condition:**

**Are you a close relative or caretaker of a person that belongs to a vulnerable group?**

- Yes
- No

**In the past did you have any mental health problem serious enough to make you seek professional health, psychotherapy or medication treatment?**

- No
- Anxiety
- Depression
- Psychosis
- Bipolar Disorder
- Other

**Are you currently under any kind of treatment for your mental state? (please check all of those that are true)**

- No
- Psychotherapy
- Antipsychotics
- Antidepressants
- Tranquilizers**/**benzodiazepines (lexotanil, xanax, tavor, etc.)

**C. THOUGHTS ABOUT COVID-19 DURING THE LOCKDOWN**

**Were you afraid that you will contract the coronavirus;**

- Never
- A little
- Moderately
- Much
- Very Much

**Do you believe that the precautions work effectively or that if you are about to contract the disease, you will contract it anyway?**

- Precautions work effectively.
- Precautions cannot protect you.

**Did the possibility that a member of your family could contract the coronavirus and die because of it made you frightened?**

- Never
- A little
- Moderately
- Much
- Very Much

**Were you afraid that in case you contracted the coronavirus, some people will step away from your life and behave to you in a different way later?**

- Never
- A little
- Moderately
- Much
- Very Much

**D. THOUGHTS ABOUT THE MEASURES TAKEN (PRECAUTIONS, LOCKDOWN, ETC.)**

**According to your opinion, during the lockdown, the amount of time that you spend outside of your house for reasons not regarding your work during this period is:**

- Minimum
- Less than humanly necessary
- Moderate**/**reasonable
- Enough
- A lot
- Excessive

**Were you locked up in the house during the lockdown period?**

- Completely
- To a high degree
- Partially
- Not at all

**According to the instructions given by WHO, it is necessary for some precautions to be taken in order to prevent the spread of the virus. Do you believe that you took adequate precautions?**

- Not at all
- A little bit
- Moderately
- Much
- Very Much

**Do you believe that that you have received sufficient information about the necessity of the measures taken?**

- Not at all
- A little bit
- Moderately
- Much
- Very Much

**E. FAMILY DURING THE LOCKDOWN**

**Did you feel the need to communicate with** **other members of your family during that period?**

- Much less
- Less
- Same
- More
- Much more

**Did you want to receive emotional support from other members of your family during that period?**

- Much less
- Less
- Same
- More
- Much more

**Were there any conflicts with the rest of your family members during that period?**

- Much less
- Less
- Same
- More
- Much more

**Had during the lockdown, the overall quality of relationships with the other members of your family changed compared to the one before the quarantine, due to COVID – 19?**

- Much worse
- Worse
- It has not changed
- A little bit better
- Much better

**During lockdown, did you manage to maintain a basic daily routine (waking up in the morning, regular meals and sleeping hours, activities) both yourself (if you live alone) or as a family?**

- Not at all
- Somehow, but not always
- Generally, yes
- Clearly follow (or adhere to) a routine

**If you have children, how difficult was it to manage their daily life and behavior during the lockdown?**

- Much more difficult than before
- Somehow more difficult but not always
- Same as always
- Somehow easier but not always
- Much easier than before

**How are your finances as a result of the outbreak?**

- Much more difficult than before
- Somehow more difficult
- Same as always
- Somehow easier
- Much easier than before

**F. ANXIETY DURING LOCKDOWN**

**I was feeling calm.**

- Not at all
- Somewhat
- Moderately so
- Very Much so

**I was feeling secure.**

- Not at all
- Somewhat
- Moderately so
- Very Much so

**I was feeling tense.**

- Not at all
- Somewhat
- Moderately so
- Very Much so

**I was feeling strained.**

- Not at all
- Somewhat
- Moderately so
- Very Much so

**I was feeling at ease.**

- Not at all
- Somewhat
- Moderately so
- Very Much so

**I was feeling upset.**

- Not at all
- Somewhat
- Moderately so
- Very Much so

**I was worrying over possible misfortunes.**

- Not at all
- Somewhat
- Moderately so
- Very Much so

**I was feeling satisfied.**

- Not at all
- Somewhat
- Moderately so
- Very Much so

**I was feeling frightened.**

- Not at all
- Somewhat
- Moderately so
- Very Much so

**I was comfortable.**

- Not at all
- Somewhat
- Moderately so
- Very Much so

**I was self-confident.**

- Not at all
- Somewhat
- Moderately so
- Very Much so

**I was feeling nervous.**

- Not at all
- Somewhat
- Moderately so
- Very Much so

**I was feeling jittery.**

- Not at all
- Somewhat
- Moderately so
- Very Much so

**I was indecisive.**

- Not at all
- Somewhat
- Moderately so
- Very Much so

**I was relaxed.**

- Not at all
- Somewhat
- Moderately so
- Very Much so

**I was feeling content.**

- Not at all
- Somewhat
- Moderately so
- Very Much so

**I was feeling worried.**

- Not at all
- Somewhat
- Moderately so
- Very Much so

**I was feeling confused.**

- Not at all
- Somewhat
- Moderately so
- Very Much so

**I was feeling steady.**

- Not at all
- Somewhat
- Moderately so
- Very Much so

**I was feeling pleasant.**

- Not at all
- Somewhat
- Moderately so
- Very Much so

**How much during the lockdown, had your emotional state changed in relation to the appearance of anxiety and insecurity compared to before the COVID-19 epidemic?**

- It got a lot worse
- It got a little worse
- Neither better nor worse
- It’s a little improved
- It has improved a lot

**G. SADNESS & DEPRESSION/MELANCHOLY DURING THE LOCKDOWN**

**I was bothered by things that usually don’t bother me.**

- Rarely or none of the time (less than 1 day)
- Some of a little of the time (1-2 days)
- Occasionally or a moderate amount of time (3-4 days)
- Most or all of the time (5-7 days)

**I did not feel like eating; my appetite was poor.**

- Rarely or none of the time (less than 1 day)
- Some of a little of the time (1-2 days)
- Occasionally or a moderate amount of time (3-4 days)
- Most or all of the time (5-7 days)

**I felt that I could not shake off the blues even with help from my family or friends.**

- Rarely or none of the time (less than 1 day)
- Some of a little of the time (1-2 days)
- Occasionally or a moderate amount of time (3-4 days)
- Most or all of the time (5-7 days)

**I felt I was just as good as other people.**

- Rarely or none of the time (less than 1 day)
- Some of a little of the time (1-2 days)
- Occasionally or a moderate amount of time (3-4 days)
- Most or all of the time (5-7 days)

**I had trouble keeping my mind on what I was doing.**

- Rarely or none of the time (less than 1 day)
- Some of a little of the time (1-2 days)
- Occasionally or a moderate amount of time (3-4 days)
- Most or all of the time (5-7 days)

**I felt depressed.**

- Rarely or none of the time (less than 1 day)
- Some of a little of the time (1-2 days)
- Occasionally or a moderate amount of time (3-4 days)
- Most or all of the time (5-7 days)

**I felt that everything I did was an effort.**

- Rarely or none of the time (less than 1 day)
- Some of a little of the time (1-2 days)
- Occasionally or a moderate amount of time (3-4 days)
- Most or all of the time (5-7 days)

**I felt full of hope about the future.**

- Rarely or none of the time (less than 1 day)
- Some of a little of the time (1-2 days)
- Occasionally or a moderate amount of time (3-4 days)
- Most or all of the time (5-7 days)

**I thought my life had been a failure.**

- Rarely or none of the time (less than 1 day)
- Some of a little of the time (1-2 days)
- Occasionally or a moderate amount of time (3-4 days)
- Most or all of the time (5-7 days)

**I felt fearful.**

- Rarely or none of the time (less than 1 day)
- Some of a little of the time (1-2 days)
- Occasionally or a moderate amount of time (3-4 days)
- Most or all of the time (5-7 days)

**My sleep was restless.**

- Rarely or none of the time (less than 1 day)
- Some of a little of the time (1-2 days)
- Occasionally or a moderate amount of time (3-4 days)
- Most or all of the time (5-7 days)

**I was happy.**

- Rarely or none of the time (less than 1 day)
- Some of a little of the time (1-2 days)
- Occasionally or a moderate amount of time (3-4 days)
- Most or all of the time (5-7 days)

**I talked less than usual.**

- Rarely or none of the time (less than 1 day)
- Some of a little of the time (1-2 days)
- Occasionally or a moderate amount of time (3-4 days)
- Most or all of the time (5-7 days)

**I felt lonely.**

- Rarely or none of the time (less than 1 day)
- Some of a little of the time (1-2 days)
- Occasionally or a moderate amount of time (3-4 days)
- Most or all of the time (5-7 days)

**People were unfriendly.**

- Rarely or none of the time (less than 1 day)
- Some of a little of the time (1-2 days)
- Occasionally or a moderate amount of time (3-4 days)
- Most or all of the time (5-7 days)

**I enjoyed life.**

- Rarely or none of the time (less than 1 day)
- Some of a little of the time (1-2 days)
- Occasionally or a moderate amount of time (3-4 days)
- Most or all of the time (5-7 days)

**I had crying spells.**

- Rarely or none of the time (less than 1 day)
- Some of a little of the time (1-2 days)
- Occasionally or a moderate amount of time (3-4 days)
- Most or all of the time (5-7 days)

**I felt sad.**

- Rarely or none of the time (less than 1 day)
- Some of a little of the time (1-2 days)
- Occasionally or a moderate amount of time (3-4 days)
- Most or all of the time (5-7 days)

**I felt that people disliked me.**

- Rarely or none of the time (less than 1 day)
- Some of a little of the time (1-2 days)
- Occasionally or a moderate amount of time (3-4 days)
- Most or all of the time (5-7 days)

**I could not get “going”.**

- Rarely or none of the time (less than 1 day)
- Some of a little of the time (1-2 days)
- Occasionally or a moderate amount of time (3-4 days)
- Most or all of the time (5-7 days)

**During the lockdown, how much has your emotional state related to the experience of joy or melancholy changed in comparison to before the COVID-19 epidemic?**

- It got a lot worse
- It got a little worse
- Neither better nor worse
- Improved a bit
- It has improved a lot

**H. PHYSICAL ACTIVITY DURING THE LOCKDOWN**

**Does exercise help you at the prevention of anxiety?**

- Not at all
- A little bit
- Moderately
- Much
- Very much

**Do you consider that exercise is important during this pandemic?**

- Not at all
- A little bit
- Moderately
- Much
- Very much

**Do you have increased the frequency and intensity of your physical workout during this pandemic and lockdown?**

- Not at all
- A little bit
- Moderately
- Much
- Very much

**How much has your physical activity been affected by this epidemic of COVID-19?**

- It decreased much
- It decreased a little
- Neither decreased, nor increased
- It increased a little
- It increased much

**I. NUTRITION DURING THE LOCKDOWN**

**During the days of the lockdown did you notice the need to eat larger amounts of food or eat more often?**

- I eat much less than I used to
- I eat bit less than I used to
- Neither more nor less
- I eat a bit more than I used to
- I eat much more than I used to

**Please mark the answer that best represents you during the period of the lockdown:**

- I was eating in a healthier way.
- My eating habits and preferences have not changed.
- I was eating in a more unhealthy way.

**Please mark the answer that best represents you during the period of the lockdown:**

- My body weight has significantly decreased (more than 2-3 kilos).
- My body weight has slightly decreased (less than 2 kilos lost).
- My body weight is stable.
- My body weight has slightly increased (less than 2 kilos put).
- My body weight has significantly increased (more than 2-3 kilos).

**J. OPINIONS ABOUT THE ORIGIN OF THE EPIDEMIC**

**Do you believe that the COVID-19 vaccine was ready even before the virus broke out and they conceal it from us for the benefit of pharmaceutical companies?**

- I don’t believe it at all
- A little bit
- Maybe
- Much
- Very much

**Do you believe that COVID-19 was created in a laboratory to be used as a biochemical weapon for the extermination of the human population?**

- I don’t believe it at all
- A little bit
- Maybe
- Much
- Very much

**Do you believe that COVID-19 is the result of 5G technology antenna?**

- I don’t believe it at all
- A little bit
- Maybe
- Much
- Very much

**Do you believe that COVID-19 appeared accidentally from human contact with animals and it was something that generally happens and was generally expected?**

- I don’t believe it at all
- A little bit
- Maybe
- Much
- Very much

**Do you believe that covid-19 has much lower mortality rate but there is misinformation and terror-inducing propaganda?**

- I don’t believe it at all
- A little bit
- Maybe
- Much
- Very much

**Do you believe that COVID-19 is a creation of the world’s powerful leaders to create a global economic crisis?**

- I don’t believe it at all
- A little bit
- Maybe
- Much
- Very much

**Do you believe that CONID-19 is a sign of divine power to destroy our planet?**

- I don’t believe it at all
- A little bit
- Maybe
- Much
- Very much

**K. INTERNET USE DURING THE LOCKDOWN**

**The information and use of the internet worry me about the issue regarding the COVID-19:**

- Not at all
- A little
- Moderately
- Much
- Very much

**Generally, most of the internet sources regarding information about COVID-19 are misinforming/misleading:**

- Not at all
- A little
- Moderately
- Much
- Very much

**Due to the conditions of the lockdown, the internet was taking up more of my time than usual:**

- Not at all
- A little more
- Moderately more
- Much more
- Too much

**How much do you use the social media while in isolation at home?**

- More than before
- The same as before
- Less than before

**During the lockdown, have you acquired internet-related habits that you did not have before (for example: created a facebook account, engaging in cybersex or gambling)?**

- Yes
- No

**L. SLEEP DURING THE LOCKDOWN**

**The quality of my sleep changed during lockdown. It was:**

- Much worse
- A little bit worse
- The same (neither worse nor better)
- A little better
- Much better

**I tended to stay up late and sleep for many hours during the day.**

- Almost never
- Rarely
- Sometimes
- Often
- Almost always

**I was taking sleeping pills to help me sleep at night during the lockdown.**

- Almost never
- Rarely
- Some times
- Often
- Almost always

**I was having dreams in which I was feeling trapped, over the period of the lockdown.**

- Almost never
- Rarely
- Some times
- Often
- Almost always

**M.** **SMOKING, ALCOHOL AND SUBSTANCES USE DURING THE LOCKDOWN**

**Smoking before the epidemic:**

- - I didn't smoke
  - I was smoking

**Alcohol use before the epidemic:**

- - I did not drink much
  - I drank a lot (more than one drink or its equivalent every day)

**Use of illegal substances before the epidemic (e.g. hashish):**

- - I did not use it
  - Occasionally and rather rarely
  - Often

**During lockdown, you smoke compared to before:**

- - More than before
  - Same as before
  - Less than before

**During lockdown, you drink alcohol compared to before:**

- - More than before
  - Same as before
  - Less than before

**While isolated at home, you use illegal substances compared to before:**

- - More than before
  - Same as before
  - Less than before

**N. SEXUALITY DURING THE LOCKDOWN**

**How would you characterize the frequency of your sexual intercourse during the lockdown?**

- Clearly inadequate
- Rather inadequate
- Neutral
- Rather satisfactory
- Clearly satisfactory

**Due to the new lockdown conditions, has your desire for sexual intercourse increased or decreased?**

- Has decreased a lot
- Has decreased slightly
- Remains the same
- Has increased slightly
- Has increased a lot

**How much pleasure and satisfaction do you get from your sex life during the lockdown?**

- Not at all
- A little bit
- Moderate
- Much
- Very much

**Do you think sex was helpful for you to deal with your daily stress and anxiety during the lockdown?**

- Not at all
- A little bit
- Moderate
- Much
- Very much

**O. THOUGHTS ABOUT DEATH DURING THE LOCKDOWN**

**Were you afraid that you are going to die?**

- Not at all
- A little bit
- Much
- Very much

**Did you ever think that it would be better if you were dead?**

- Not at all
- A little bit
- Much
- Very much

**Were you thinking that it is a wonderful thing that you are alive?**

- Not at all
- A little bit
- Much
- Very much

**Have you ever felt that it’s worth living?**

- Not at all
- A little bit
- Much
- Very much

**Were you thinking of harming yourself physically?**

- Not at all
- A little bit
- Much
- Very much

**Were you often thinking of committing suicide if you had the chance?**

- Not at all
- A little bit
- Much
- Very much

**Did you make plans concerning the method to use in order to end your life?**

- Not at all
- A little bit
- Much
- Very much

**I was thinking of committing suicide, but I wouldn’t do it.**

- Not at all
- A little bit
- Much
- Very much

**Were you enjoy your life?**

- Not at all
- A little bit
- Much
- Very much

**Were you feeling tired from your life?**

- Not at all
- A little bit
- Much
- Very much

**How much had your tendency to think about death and/or suicide changed, compared to before the outbreak of COVID-19?**

- Very much increased
- Increased a bit
- Neither increased, nor decreased
- Decreased a bit
- Very much decreased

**Have you ever hurt yourself in any way deliberately, during your whole life so far?**

- Never
- Once
- 2-3 times
- Many times

**Have you ever attempted suicide, during your whole life so far?**

- Never
- Once
- 2-3 times
- Many times

**P. SPIRITUALITY AND PSYCHOLOGICAL REFECTIONS**

**Over the period of lockdown, my religious/spiritual inquiries have been increased.**

- Not at all.
- A little bit
- Much
- Very Much
